# Expression of S100A4, ephrin-A1 and osteopontin in non-small cell lung cancer

**DOI:** 10.1186/1471-2407-12-333

**Published:** 2012-08-01

**Authors:** Ane Kongsgaard, Marius Lund-Iversen, Gisle Berge, Odd Terje Brustugun, Steinar K Solberg, Gunhild M Mælandsmo, Kjetil Boye

**Affiliations:** 1 Department of Tumor Biology, Institute for Cancer Research, The Norwegian Radium Hospital, Oslo University Hospital, PO Box 4953, Nydalen, NO-0424, Oslo, Norway; 2 Department of Pathology, The Norwegian Radium Hospital, Oslo University Hospital, PO Box 4953, Nydalen, NO-0424, Oslo, Norway; 3 Department of Oncology, The Norwegian Radium Hospital, Oslo University Hospital, PO Box 4953, Nydalen, NO-0424, Oslo, Norway; 4 Department of Cardiovascular and Thoracic Surgery, Rikshospitalet, Oslo University Hospital, PO Box 4953, Nydalen, NO-0424, Oslo, Norway; 5 Department of Pharmacy, Faculty of Health Sciences, University of Tromsø, 9037, Tromsø, Norway

**Keywords:** S100A4, Ephrin-A1, Osteopontin, NSCLC, Immunohistochemistry

## Abstract

**Background:**

The metastasis-promoting protein S100A4 induces expression of ephrin-A1 and osteopontin in osteosarcoma cell lines. The aim of this study was to investigate S100A4-mediated stimulation of ephrin-A1 and osteopontin in non-small cell lung cancer (NSCLC) cell lines, and to characterize the expression of these biomarkers in primary tumor tissue from NSCLC patients.

**Methods:**

Four NSCLC cell lines were treated with extracellular S100A4, and ephrin-A1 and osteopontin expression was analyzed by real time RT-PCR and Western blotting. Immunohistochemical staining for S100A4, ephrin-A1 and osteopontin was performed on tissue microarrays containing primary tumor samples from a cohort of 217 prospectively recruited NSCLC patients, and associations with clinicopathological parameters were investigated.

**Results:**

S100A4 induced ephrin-A1 mRNA and protein expression in adenocarcinoma, but not in squamous carcinoma cell lines, whereas the level of osteopontin was unaffected by S100A4 treatment. In primary tumors, moderate or strong immunoreactivity was observed in 57% of cases for cytoplasmic S100A4, 46% for nuclear S100A4, 86% for ephrin-A1 and 77% for osteopontin. Interestingly, S100A4 expression was associated with ephrin-A1 also in vivo, but there was no association between S100A4 and osteopontin. Expression levels of S100A4 and ephrin-A1 were significantly higher in adenocarcinomas compared to other histological subtypes, and S100A4-positive tumors were smaller and more differentiated than tumors without expression.

**Conclusions:**

Our findings suggest that S100A4, ephrin-A1 and osteopontin are involved in the biology of NSCLC, and further investigation of their potential use as biomarkers in NSCLC is warranted.

## Background

Lung cancer is one of the most frequently occurring malignancies, and the leading cause of cancer-related death in men and the second leading cause in women [1]. Non-small cell lung cancer (NSCLC) accounts for approximately 85% of all lung cancer cases, with adenocarcinoma, squamous cell carcinoma and large cell carcinoma as the main histological subtypes. Surgical resection or radiotherapy have curative potential, and in Norway the 5-year survival among patients with early stage disease who undergo complete surgical resection is approximately 65% [2]. Even for stage I patients there is a significant risk of relapse, and NSCLC carries one of the most dismal outcomes for stage I disease among all tumor types [3]. Clearly, there is an urgent need for more effective treatment as well as improved classification algorithms to identify patients at increased risk of relapse.

NSCLC patients who undergo curatively intended surgery are stratified according to TNM (tumor-node-metastasis) staging, and based on this patients are selected for adjuvant therapy. However, tumors within the same disease stage are biologically heterogeneous and behave differently, and identification of biomarkers could enable further subclassification of patients and thereby a more accurate prediction of prognosis. Furthermore, the increased use of targeted therapies in NSCLC requires improved knowledge about molecular alterations in the tumor cells to facilitate therapeutic decisions.

One potentially interesting molecular marker is S100A4, a member of the S100 family of calcium binding proteins. S100A4 is localized in the cytoplasm, nucleus and extracellular space and has multiple biological functions including regulation of angiogenesis and stimulation of motility and invasion. S100A4 promotes metastasis in several experimental animal models and is associated with patient outcome in a variety of cancer types [4]. In lung cancer, experimental models have shown that there is an association between S100A4 expression and motile and invasive abilities, and that suppression of S100A4 results in reduced metastatic potential [5,6].

Several studies have investigated S100A4 protein expression in NSCLC, with the percentage of positive cases ranging from 20-84% [7-11]. In general, S100A4 is not expressed in normal lung epithelium [7], whereas a variety of cells in the tumor microenvironment are S100A4-positive, including lymphocytes, fibroblasts and smooth muscle cells [9,10]. In some examinations, S100A4 expression has been shown to be associated with pT stage and poor patient outcome [9], while other studies have failed to demonstrate a prognostic role for S100A4 in NSCLC [7,8].

Ephrin-A1 (Eph receptor interacting protein-A1) is a cell surface protein which can act as a ligand for several of the Eph receptor tyrosine kinases, such as EphA2, EphA3 and EphA4 [12]. Ephrin-A1 is involved in multiple biological processes, including tumor angiogenesis [13,14], cell motility [15] and metastasis [16,17]. To our knowledge, the role of ephrin-A1 in lung cancer has not been investigated, and based on its pro-metastatic functions in other types of cancer, characterization of the expression in NSCLC would be of substantial interest.

Osteopontin, a member of the small integrin-binding ligand N-linked glycoprotein (SIBLING) family, is a secreted chemokine-like multifunctional protein. Biological processes regulated by osteopontin include adhesion, migration, invasion, proteolysis, enhanced cell survival and angiogenesis [18,19], and several studies have shown an association between high osteopontin expression and poor patient outcome in NSCLC [20-22].

Our group has previously shown that extracellular S100A4 induces the expression of ephrin-A1 and osteopontin in osteosarcoma cell lines [18,23]. Based on the reported biological effects of ephrin-A1 and osteopontin, S100A4-induced expression of these molecules may be one of several mechanisms by which S100A4 promotes tumor progression. The aim of the present study was to investigate whether S100A4 induces expression of ephrin-A1 and osteopontin in NSCLC, and to characterize the expression of these molecular markers in primary tumor tissue from prospectively recruited patients undergoing curative surgery for NSCLC. Furthermore, associations between expression of these proteins and clinical and histopathological parameters were investigated.

## Methods

### Cell culture and treatment

The human lung adenocarcinoma cell line EKVX was established at Department of Tumor Biology, The Norwegian Radium Hospital, Oslo University Hospital. The adenocarcinoma cell line A549 and the squamous cell carcinoma cell lines HTB-182 (NCI-H520) and SW900 (HTB-59), were purchased from the American Type Culture Collection (Rockville, MD, USA). Recombinant human S100A4 protein was produced as described previously [18]. Cells were cultivated in RPMI 1640 (Lonza, Verviers, Belgium), supplemented with 8.5% fetal bovine serum (PAA Laboratories, Pasching, Austria), 20 mM Hepes buffer (Lonza) and 2 mM GlutaMAX (Gibco, Invitrogen, Oslo, Norway). All cell cultures were routinely tested for Mycoplasma infection. The identity of the cell lines were determined by STR profiling using Powerplex 16 (Promega, Madison, WI, USA). For cell culture experiments, subconfluent cell cultures were detached with Versene EDTA (Lonza), and 1 × 10^6^ cells were seeded in T25 flasks and grown overnight. The following day, the culture medium was replaced with medium with or without recombinant human S100A4 protein (2 μg/ml or 10 μg/ml) and further incubated for 6 or 24 hours. Cells were harvested by Tri-reagent (Ambion, Applied Biosystems Europe, Oslo, Norway) for RNA isolation, and by scraping for preparation of cell lysates.

### Real time RT-PCR

One microgram total RNA was reverse transcribed using the iScript RT kit (Bio-Rad, Hercules, CA, USA). Gene expression levels were examined by quantitative real-time reverse transcription PCR (qPCR) as described in Boye *et al.*[23] for ephrin-A1 and Berge *et al.*[18] for osteopontin. The PCR threshold cycle number (Ct) was used to calculate the relative expression of each gene normalized to the expression of an endogenous control gene as follows: 2^−ΔCt^, where ΔCt = Ct_gene_ – Ct_control gene_.

### Western blot analysis

Western blotting was performed as described previously [18]. Antibody against ephrin-A1 was obtained from Santa Cruz Biotechnology (sc-911, Santa Cruz Biotechnology, Santa Cruz, CA, USA).

### Patient cohort

Primary tumor samples were prospectively collected from 244 patients with assumed or verified NSCLC who were considered operable and underwent curatively intended surgical resection at Rikshospitalet, Oslo University Hospital, Oslo, Norway between March 2006 and April 2010. Following surgery, resected tissue was processed for routine histopathological examination. The study was approved by the Regional Ethics Committee (S-06402b), and all patients were informed and signed a written consent. Twenty-seven patients were excluded from the study for the following reasons: histology other than NSCLC (carcinoid (12), small cell lung cancer (4), lung metastases from other primary cancer (7)) and withdrawal of consent (4). The study population thus included 217 patients with histologically verified primary NSCLC. Histological examination of all tissue specimens was performed by experienced pathologists, and the histopathological parameters were retrieved from the pathology reports. The tumors were staged according to the International Association for the Study of Lung Cancer (IASLC), TNM 7. The histological subtypes were classified according to WHO criteria, with adenocarcinoma, squamous cell carcinoma and large cell carcinoma as the three main types. Bronchioalveolar carcinomas were included in the adenocarcinoma group, constituting 4.5% of these tumors. Seven patients received neoadjuvant chemotherapy and/or radiation therapy due to the following reasons: pancoast tumor, N2 disease and for downstaging of a primarily inoperable tumor. The patients´ tobacco use was registered and divided into three groups; current smoker, former smoker or never smoker. Never smoker was defined as never having smoked on a regular basis, and former smoker was defined as having quit smoking at least one year before inclusion in the study.

### Tissue microarray (TMA) construction

TMA sections were constructed using a tissue arrayer instrument (Beecher Instruments, Silver Springs, MD, USA). Formalin-fixed tumor tissue from 206 patients was available for TMA construction. The most representative tumor areas in each donor block were selected by an experienced pathologist and marked on hematoxylin-eosin stained sections. From corresponding blocks, one mm core biopsies were obtained from at least two different tumor-rich areas, and two additional cores were selected from adjacent normal lung tissue. The cores were inserted directly into the recipient paraffin block in a grid arrangement, and one slide from each prepared TMA block was stained with hematoxylin-eosin for tumor tissue confirmation.

### Immunohistochemistry

The TMA sections were immunostained for S100A4 and osteopontin using the EnVision^TM^ FLEX + detection system from Dako (Dako, Glostrup, Denmark). Dako PT link was used for deparaffinization and heat-induced epitope retrieval. Sections were preheated in Dako EnVision FLEX + Target Retrieval Solution, High pH and rinsed in Dako wash buffer according to the manufacturer´s instructions. Thereafter, endogenous peroxidase activity was blocked for 5 minutes using 0.03% H_2_O_2_, sections were washed twice in Dako wash buffer and incubated for 30 minutes with primary antibody at room temperature. After an additional washing step, slides were incubated with secondary antibody (HRP-labelled polymer conjugated to anti-mouse or anti-rabbit immunoglobulins) for 30 minutes at room temperature. After new washing, sections were incubated for 10 minutes in DAB (diaminobenzidine). Finally the sections were rinsed twice in water before counterstaining with hematoxylin and mounting in Diatex. The following primary antibodies were used: mouse monoclonal anti-S100A4 (20.1) [24] diluted 1:300 and rabbit polyclonal anti-osteopontin diluted 1:300 (Rb-9097, Thermo Fisher Scientific, Fremont, CA, USA). Ephrin-A1 immunostaining was done using the EnVision + system from Dako (Dako) as follows: TMA slides were deparaffinized with xylene, rehydrated through graded ethanol solutions and rinsed in distilled water. For antigen retrieval, tissue sections were preheated in a microwave oven at 100 ° C for 15 minutes in Tris/EDTA solution, left in the buffer for 10 minutes after boiling, rinsed in distilled water and in Dako wash buffer. The rest of the procedure was performed as described for S100A4 and osteopontin. The primary antibody used was rabbit polyclonal anti-ephrin-A1 diluted 1:300 (sc-911, Santa Cruz Biotechnology). Sections from colorectal tumor tissue, ovarian tissue and cervical portio biopsy tissue known to express high amounts of S100A4, osteopontin and ephrin-A1, respectively, were used as positive controls.

### Evaluation of immunohistochemistry

All immunostained sections were evaluated by two investigators (A.K.R and K.B for S100A4, and A.K.R and M.L-I for ephrin-A1 and osteopontin), and discrepancies were resolved by consensus. Immunohistochemical expression was evaluated without knowledge on the corresponding clinicopathological parameters. In nine cases staining was not evaluable due to lack of representative tumor material. S100A4 immunoreactivity was apparent as both cytoplasmic and nuclear staining, and these were recorded as individual variables (S100A4c and S100A4n, respectively). The samples were scored using a 0–3 scale according to staining intensity, with 0 denoting negative (no staining), 1 denoting weak staining, 2 intermediate staining and 3 strong staining. For nuclear staining, the fraction of positively stained nuclei were estimated (0 = 0%, 1 = < 1%, 2 = 1 – 10%, 3 = 11 – 33%, 4 = 34 – 66% and 5 = 67 – 100%). All samples with >10% stained nuclei (score ≥ 3) were considered positive, and grouped according to staining intensity (implying that a sample with 50% stained nuclei and intensity score 2 would be given 2 as a final score). Osteopontin and ephrin-A1 showed less variation in staining intensity than S100A4, and differentiating between weak and intermediate staining was difficult. Consequently, osteopontin and ephrin-A1 immunoreactivity was scored according to a 0–2 scale, with 0 defined as negative (no staining), 1 as intermediate staining and 2 as strong staining. The percentage of positive tumor cells was not evaluated for S100A4c, ephrin-A1 and osteopontin because there was uniform staining of the tumor cells in the vast majority of cases, and thus the estimation of the fraction of stained cells provided no additional information. For all three biomarkers, the dominant staining intensity was scored. Furthermore, at least two cores from different tumor areas of the same specimen were included in the TMA, and the staining intensity was highly similar in the analysed cases.

### Statistical analysis

Statistical analyses were performed using SPSS version 16.0 (SPSS Inc., Chicago, IL, USA). Associations between expression of S100A4, ephrin-A1 and osteopontin, and associations between immunohistochemical expression and clinicopathological variables were examined using two-tailed Fisher´s exact test or linear by linear association chi-square test. For the RT-PCR experiments, and to compare the mean tumor size of the S100A4 negative and positive tumors, two-tailed Student´s *t*-test was used. P values < 0.05 were considered statistically significant.

## Results

### Induction of ephrin-A1 and osteopontin expression by extracellular S100A4 in NSCLC cell lines

**Figure 1:**
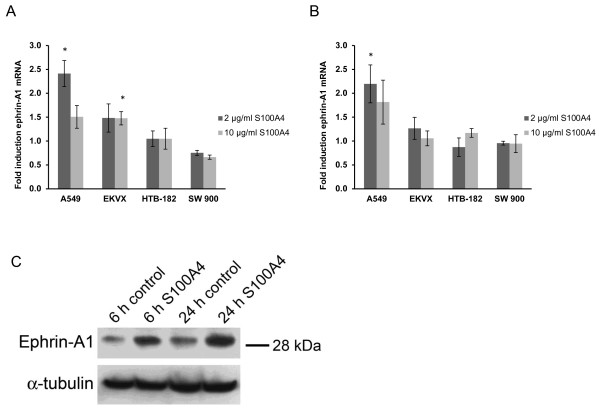
**Induction of ephrin-A1 expression by extracellular S100A4 in NSCLC cell lines.A** and **B**) Fold induction of ephrin-A1 mRNA in NSCLC cell lines treated with 2 μg/ml and 10 μg/ml S100A4 for 6 hours (A) and 24 hours (B) compared to untreated control cells. Bars represent mean fold induction, n = 3, *p < 0.05. (**C**) Western blot of total cell lysates from A549 cells treated with 2 μg/ml S100A4 for 6 or 24 hours as indicated. Membranes were stained with anti-ephrin-A1, and α-tubulin was used as a loading control.

To investigate if S100A4 stimulates expression of ephrin-A1 and osteopontin in NSCLC, two adenocarcinoma and two squamous cell carcinoma cell lines were treated with 2 and 10 μg/ml recombinant S100A4 for 6 and 24 hours. Ephrin-A1 and osteopontin mRNA expression was analyzed by RT-PCR. As depicted in Figure 1A and 1B extracellular S100A4 induced expression of ephrin-A1 in adenocarcinoma cell lines. In A549 cells, treatment with 2 μg/ml S100A4 increased the ephrin-A1 mRNA levels 2.4 fold (p = 0.006) and 2.2 fold (p = 0.03) after 6 h and 24 hours, respectively. The induction was also confirmed at the protein level as shown in Figure 1C. In EKVX cells, ephrin-A1 mRNA increased 1.5 fold after treatment with both 2 μg/ml and 10 μg/ml S100A4 for 6 hours (p = 0.17 and 0.03, respectively). The squamous cell carcinoma cell lines HTB-182 and SW900 did not show any significant regulation of ephrin-A1 upon S100A4 treatment. Furthermore, extracellular S100A4 did not influence osteopontin levels in any of the cell lines tested (data not shown).

### Clinicopathological characteristics of the patient cohort

Next, we wanted to examine the expression of S100A4, ephrin-A1 and osteopontin in NSCLC tumor tissue. At the time of surgery, 217 patients with histologically verified NSCLC who underwent curatively intended surgical resection were prospectively included. The clinical and histopathological parameters of the study cohort are summarized in Table 1. The cohort included 116 (53%) males and 101 (47%) females, with a combined median age of 66 years (range 39–83 years). The most common histological type was adenocarcinoma (61%), followed by squamous cell carcinoma (28%) and large cell carcinoma (11%). The majority of the patients were in the early disease stages, with 63% in TNM stage I and 19% in TNM stage II.

**Table 1:** Clinicopathological characteristics of the patient cohort

**Parameter**		**Patients**	
		Number	Percent
Gender	Male	116	53
	Female	101	47
Age at surgery	< 65 years	100	46
	> 65 years	117	54
Histology	Adenocarcinoma (incl. BAC)	132	61
	Squamous cell carcinoma	60	28
	Large cell carcinoma	25	11
Differentiation	G1 (well differentiated)	18	9
	G2 (moderately differentiated)	138	69
	G3 (poorly differentiated)	45	22
	Missing	16	
pTNM	I	135	63
	II	42	19
	III	35	16
	IV	4	2
	Missing*	1	
pT	pT1	68	31
	pT2	120	56
	pT3	17	8
	pT4	11	5
	Missing*	1	
pN	0	157	73
	1	38	17
	2	21	10
	Missing*	1	
pM	0	212	98
	1	4	2
	Missing*	1	
Tumor size	< 2.0 cm	58	27
	2.1-3.0 cm	62	29
	3.1-5.0 cm	65	30
	5.1-7.0 cm	23	10
	> 7.0 cm	8	4
	Missing*	1	
Surgery	Lobectomy	147	68
	Pulmectomy	26	12
	Wedge resection	17	8
	Bilobectomy	17	8
	Other	10	4
Tobacco use	Current smoker	74	34
	Former smoker	129	60
	Never smoker	14	6

* TNM stage missing in one patient due to exploratory surgery.

### Expression of S100A4, ephrin-A1 and osteopontin in primary NSCLC

**Figure 2:**
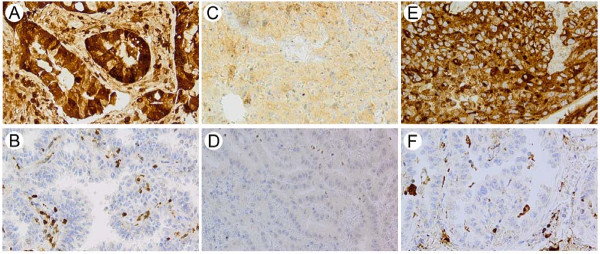
**Expression of S100A4, ephrin-A1 and osteopontin in primary NSCLC.** Representative photomicrographs of NSCLC specimens stained with anti-S100A4 (**A** and **B**), anti-ephrin-A1 (**C** and **D**) and anti-osteopontin (**E** and **F**). A, C and E demonstrate strong immunoreactivity, whereas negative staining is shown in B, D and F.

Of the 217 included patients, evaluable tumor tissue was present in 196 cases. S100A4, ephrin-A1 and osteopontin all displayed weak positive expression in normal alveolar and bronchial epithelial cells. In tumor stroma most inflammatory cells were stained, and the positivity was especially prominent in macrophages. However, there was variation in staining intensity from weak to strong for all three markers. The staining of normal cells was not systematically assessed, and thus not further analyzed. An overview of the immunohistochemical expression of S100A4, ephrin-A1 and osteopontin in tumor cells is presented in Table 2, and representative microscope images are shown in Figure 2. S100A4 immunoreactivity was apparent both in the cytoplasm and in the nucleus. Twenty percent showed strong cytoplasmic staining, 37% were moderately positive, 41% weakly positive and 2% negative. For nuclear staining 20% were strongly positive, 26% moderately positive, 34% weakly positive and 20% were negative. Ephrin-A1 was expressed both in the cytoplasm and on the cell membrane, and 14% of the tumors were strongly positive and 72% moderately positive. Osteopontin immunoreactivity was detectable as granular cytoplasmic staining in the tumor cells, and occasional staining of tumor cell nuclei was also observed. In total, 77% of the tumors were positive for osteopontin, with 12% of cases showing strong staining and 65% displaying moderate staining.

**Table 2:** Immunohistochemical expression of S100A4, ephrin-A1 and osteopontin

		**Number**	**Percent**
S100A4c	Negative	3	2
	Weak	81	41
	Moderate	73	37
	Strong	39	20
S100A4n	Negative	39	20
	Weak	66	34
	Moderate	52	26
	Strong	39	20
Ephrin-A1	Negative	28	14
	Moderate	142	72
	Strong	26	14
Osteopontin	Negative	45	23
	Moderate	128	65
	Strong	23	12

### Association of S100A4, ephrin-A1 and osteopontin with histology

Interestingly, the expression levels of S100A4c, S100A4n and ephrin-A1 were significantly higher in adenocarcinomas compared to the squamous and large cell tumors (Table 3). In fact, for both S100A4c and S100A4n 29% of the adenocarcinomas showed strong staining, compared to only 6% of the squamous cell carcinomas, and 4% of the large cell tumors (p = 0.001). Eighteen percent of the adenocarcinomas showed strong positive staining for ephrin-A1, compared to 6% and 4% of the squamous and large cell tumors, respectively (p = 0.004). The expression of osteopontin was evenly distributed in the different histological subtypes.

**Table 3:** Associations between immunohistochemical expression, histology and tumor size

		**Histological subtype**				**Tumor size**		
		**ADC (n = 123)**	**SCC (n = 49)**	**LCC (n = 24)**	p value*	**≤ 3.0 cm (n = 1 9)**	**≥ 3.1 cm (n = 87)**	p value*
S100A4c	negative/weak	34%	57%	58%		36%	51%	
	moderate	37%	37%	38%		38%	36%	
	strong	29%	6%	4%	0.001	26%	13%	0.01
S100A4n	negative	13%	33%	29%		18%	22%	
	weak	32%	34%	38%		29%	40%	
	moderate	26%	27%	29%		27%	25%	
	strong	29%	6%	4%	0.001	26%	13%	0.04
Ephrin-A1	negative	11%	29%	4%		15%	14%	
	moderate	71%	65%	92%		70%	75%	
	strong	18%	6%	4%	0.004	15%	11%	0.79
Osteopontin	negative	23%	21%	25%		26%	20%	
	moderate	67%	61%	66%		62%	69%	
	strong	10%	18%	9%	0.61	12%	11%	0.54

* p value calculated by Fisher´s exact test or linear by linear association test as appropriate.

### Association of S100A4, ephrin-A1 and osteopontin with other clinicopathological parameters

A highly significant association between S100A4c and tumor size was observed. Fifty-one percent of the tumors with a diameter of more than 3.0 cm displayed weak or negative cytoplasmic staining for S100A4, compared to 36% for tumors less than 3.0 cm (Table 3). For tumors with strong cytoplasmic S100A4 immunoreactivity, the mean tumor diameter was 2.6 cm, whereas the mean diameter for S100A4c-negative or weakly stained tumors was 3.4 cm (p = 0.02, independent samples *t*-test). S100A4c staining did also vary relative to tumor differentiation, as 24% of the well differentiated (grade 1) tumors showed strong positive staining, compared to only 7% of the poorly differentiated tumors (p = 0.05). The associations between S100A4, ephrin-A1 and osteopontin and other clinicopathological parameters are summarized in Additional file 1. Furthermore, we performed analyses including only the cases with adenocarcinoma histology (n = 123, Additional file 2). Interestingly, we found that S100A4c expression was related to pTNM stage, with the highest levels of S100A4 found in stage I patients (p = 0.04). In addition, an inverse association was found between S100A4 staining and lymph node metastasis (pN status) (p = 0.04). There was also an association between S100A4c expression and smoking habits in terms of packyears (the number of packs of cigarettes smoked per day multiplied by the number of years the person has smoked); the patients who had smoked many packyears tended to show strong positive S100A4c staining (p = 0.02). In principal, the same associations were found between nuclear S100A4 expression and the mentioned clinicopathological parameters both in the whole patient cohort, and when analyzing the adenocarcinoma group separately. However, in the adenocarcinoma group we also found that S100A4n staining was inversely associated with pT stage. In fact, 7% of patients in pT stage 1 were negative for S100A4n, compared to 37% who had strong S100A4n staining (p = 0.04, Additional file 2). No statistically significant associations were found between immunohistochemical expression of S100A4, osteopontin or ephrin-A1 and other clinicopathological parameters (Additional file 1).

### Associations between immunohistochemical expression of S100A4, ephrin-A1 and osteopontin

In line with the in vitro data, S100A4 expression both in the cytoplasm and nucleus was associated with ephrin-A1 expression (Table 4; p = 0.02 and 0.06, respectively). In more detail, we observed that 64% of S100A4c negative and weakly stained tumors were ephrin-A1-negative, whereas only 27% were strongly positive for ephrin-A1. Furthermore, among tumors that were negative for nuclear S100A4 staining, 40% were negative for ephrin-A1, whereas 19% were ephrin-A1 strongly positive. There was no association between S100A4 and osteopontin expression. As expected, there was a highly significant association between the expression of cytoplasmic and nuclear S100A4 (p < 0.001) and among the S100A4c negative cases, none displayed nuclear staining. When selecting only the cases with adenocarcinoma histology we found a significant association between ephrin-A1 and osteopontin expression (p = 0.005), but this association was weaker (p = 0.06) when including all patients.

**Table 4:** Associations between the expression of S100A4, ephrin-A1 and osteopontin

	**S100A4c**	**S100A4n**	**Ephrin-A1**	**Osteopontin**
S100A4c		< 0.001	0.02	0.72
S100A4n	< 0.001		0.06	0.95
Ephrin-A1	0.02	0.06		0.06
Osteopontin	0.72	0.95	0.06	

* p value calculated by linear by linear association test.

## Discussion

In the present study we have demonstrated that extracellular S100A4 stimulates the expression of ephrin-A1 in NSCLC cell lines. Furthermore, we have characterized the expression of S100A4, ephrin-A1 and osteopontin in primary tumors from 217 NSCLC patients, and investigated the associations between these biomarkers and conventional clinicopathological parameters. Our group has previously shown that extracellular S100A4 induces the expression of ephrin-A1 and osteopontin in osteosarcoma cell lines by activating the transcription factor NF-κB [18,23]. Based on these results, we wanted to investigate whether S100A4-mediated induction of ephrin-A1 and osteopontin also occurs in NSCLC cell lines. Interestingly, we observed that S100A4 was able to induce expression of ephrin-A1 both at the mRNA and protein level in adenocarcinoma, but not in squamous cell carcinoma cell lines. However, no S100A4-mediated stimulation of osteopontin expression was found in any of the cell lines tested. Importantly, a significant association was also found between expression of S100A4 and ephrin-A1 in primary tumor samples from NSCLC patients, indicating that S100A4 stimulates ephrin-A1 expression both in vivo and in vitro.

We found high expression of ephrin-A1 in 13% and intermediate expression in 72% of the tumors, and the fact that ephrin-A1 is expressed in the majority of the samples may suggest that this protein plays an important biological role in NSCLC. However, ephrin-A1 was not associated with any of the clinicopathological parameters apart from histological type. Interestingly, we found that adenocarcinomas had a higher percentage of S100A4 and ephrin-A1 positivity compared to squamous and large cell tumors, and this finding is in keeping with that of previous studies on S100A4 [7-9] and ephrin-A1 [25]. The histological subclasses of NSCLC differ not only in their presentation in different regions of the lung and in outcome [26], but also in molecular characteristics and thereby in response to targeted therapies [27]. Consequently, the differences in expression patterns of the protein markers between the adenocarcinomas and squamous cell carcinomas in this study are not surprising.

Expression of S100A4 in surgically resected NSCLC specimens has previously been investigated in several studies [7-11], and the percentage of S100A4 positive cases in these studies range from 20-84%. In our study, intermediate or strong cytoplasmic expression of S100A4 was observed in 57% of the cases, which is comparable to the previous investigations. For osteopontin, high expression was found in 77% of the tumors, whereas in previous studies in NSCLC, osteopontin immunoreactivity range from 38–67% [20,21,28-30]. In contrast to previous reports, where an association between high expression and squamous cell carcinoma has been described [28,30], we did not find any significant associations between osteopontin expression and conventional clinicopathological parameters.

Possible explanations for the contradicting results for both S100A4 and osteopontin could be that different antibodies, different immunohistochemical staining techniques and different scoring systems were used. In the present study we have used immunohistochemical staining of tissue microarrays. A potential disadvantage with the use of TMA is the possibility that small tissue cores do not adequately represent the tumor, especially in cases with intratumoral heterogeneity. To evaluate whether the expression patterns of the protein markers on the small TMA cores were representative for the whole tumor, we immunostained seven whole sections with the same antibodies. The staining intensity of S100A4 and ephrin-A1 was generally homogenous across the sections, indicating that the obtained results are indeed representative of the whole tumor section. For osteopontin, however, some intratumor heterogeneity was observed. Also of importance, the majority of the mentioned studies have been retrospectively conducted, and the patient cohorts may therefore be biased. Our cohort was prospectively recruited, and the distribution of gender and age at surgery corresponds well with data from The Norwegian Association for Cardiothoracic Surgery. Thus, we believe that this patient population can be considered representative for patients with early stage NSCLC undergoing primary surgery in Norway.

S100A4 expression was associated with small tumor size and high degree of differentiation, and when analyzing the adenocarcinomas separately, significant inverse associations between S100A4 expression and lymph node metastasis as well as pTNM stage were found. Given that S100A4 in general is associated with poor prognosis and promotes metastasis in a number of tumor types [4], this result was rather unexpected. Our results are also in contrast to other investigations in NSCLC where S100A4 expression was associated with high TNM stage and poor outcome [9-11]. Importantly, in our cohort of prospectively recruited patients S100A4 expression was associated with several parameters that each reflects a less aggressive phenotype, suggesting that the observed result could be of clinical relevance, but further studies are required to clarify this issue.

How might we explain the unexpected result that S100A4 is associated with a non-aggressive phenotype in NSCLC? One of the most important biological functions contributing to S100A4-induced metastasis is increased cell migration and invasive capacity. However, induction of S100A4 has also been shown to decrease motility and invasiveness, such as in squamous cell carcinoma [31], and down-regulation of S100A4 in astrocytes increased their migratory capacity in vitro [32]. Furthermore, certain lines of evidence suggest that S100A4 may have tumor suppressor functions in the lung. S100A4 knockout mice, that were otherwise phenotypically normal, were prone to spontaneous tumor development, and the most frequent tumor observed was carcinoma of the lung [33]. Taken together, these results indicate that the biological function of S100A4 is cell type-dependent, and possibly, S100A4 may not play a pro-metastatic role in all tumor types. One might also speculate that S100A4 could inhibit tumor progression in the early stages of NSCLC development, while promoting metastasis at later disease stages, similar to the cytokine transforming growth factor β [34].

Moreover, our findings suggest that S100A4-induced expression of ephrin-A1 may be one mechanism by which S100A4 mediates its biological functions. If so, one should assume that similar functions are attributed to both proteins, and interestingly ephrin-A1 stimulates both cellular motility [15], angiogenesis [13,14] and metastasis [35], features that are also associated with S100A4 [4]. However, seemingly contradictory results have been reported for ephrin-A1, and overexpression of ephrin-A1 or treatment with ephrin-A1-Fc (soluble recombinant ephrin-A1 fused to the Fc portion of IgG) has been shown to inhibit invasiveness and reduce tumor growth in bladder, pancreatic and gastric cancer, and in malignant mesothelioma [36-40]. In addition, ephrin-A1-Fc was found to inhibit tumor growth and migration in NSCLC cells [41]*.* Ephrin-A1 is supposed to act as a tumor suppressor through its preferred receptor EphA2 [25] which is overexpressed in NSCLC [41]*.* Similar to its ligand, the role of EphA2 in cancer is somewhat conflicting. Increased expression is associated with poor clinical outcome in several tumor types, including NSCLC [3,25,42,43]. However, EphA2 can also act as a tumor suppressor [43], and recently, high expression of both EphA2 and ephrin-A1 was found to be related to favorable prognostic factors in stage I NSCLC patients [25]. Based on our findings that S100A4 is associated with small tumor size and a less aggressive phenotype, one might speculate that S100A4-mediated induction of ephrin-A1 could be implicated in reduced tumor growth and invasiveness in NSCLC. However, ephrin-A1 expression was not associated with tumor size, differentiation or tumor stage, indicating that at least these S100A4-associated features are independent of ephrin-A1. Overall, these results suggest that ephrin-A1 plays an important role in tumor progression, but the exact function is complex, cell-type dependent and most likely relies on many factors, including its preferred receptor EphA2 [44]. Furthermore, the role of ephrin-A1 as a biomarker still remains elusive, and especially in NSCLC further studies are certainly required.

## Conclusions

We have shown that in the present cohort of NSCLC patients S100A4-positive tumors were smaller and more differentiated than tumors without expression. It will be of great interest to examine whether the observed association between S100A4 expression and clinicopathological parameters also influence on patient outcome, and this will be investigated when follow-up data are available. Furthermore, we have demonstrated that S100A4 induces expression of ephrin-A1 in lung adenocarcinoma cell lines, and that the expression of these potential biomarkers is significantly associated in the primary tumor samples. Finally, our findings contribute to an increased understanding of the molecular characteristics of NSCLC, which hopefully will foster improvements in diagnostics, therapeutic decisions and the development of novel therapies.

## Competing interests

The authors declare that they have no competing interests.

## Authors’ contributions

AK conceived the study, carried out the cell culture experiments, evaluated immunostained sections, performed data analysis and wrote the manuscript. ML-I evaluated immunostained sections. GB performed real time RT-PCR analyses and Western blotting. OTB and SKS provided patient material and patient data. GMM conceived the study and participated in writing the manuscript. KB conceived the study, evaluated immunostained sections, participated in data analysis and manuscript drafting. All authors read and approved the final manuscript.

## Pre-publication history

The pre-publication history for this paper can be accessed here:

http://www.biomedcentral.com/1471-2407/12/333/prepub

## Supplementary Material

Additional file 1 Associations between clinicopathological parameters and expression of S100A4, ephrin-A1 and osteopontin. Click here for file

Additional file 2 Associations between immunohistochemical expression of cytoplasmic and nuclear S100A4 and selected clinicopathological parameters in adenocarcinomas. Click here for file
