# Supplementary material for: Expression of S100A4, ephrin-A1 and osteopontin in non-small cell lung cancer
Source: BMC Cancer. 2012 Aug 1;12:333. doi: 10.1186/1471-2407-12-333 (PMC3458900; doi:10.1186/1471-2407-12-333)
Supplement: Additional file 1 — Associations between clinicopathological parameters and expression of S100A4, ephrin-A1 and osteopontin. [file 1471-2407-12-333-S1.pdf]

Additional file 1

Associations between clinicopathological parameters and expression of S100A4, ephrin-A1 and osteopontin

Parameter		Patients		S100A4c	S100A4n	Ephrin A1	Osteopontin
		Number	Percent	p value	p value	p value	p value
Gender	Male	116	53	0.95	0.62	0.59	0.15
	Female	101	47				
Age at surgery	< 65 years	100	46	0.28	0.38	0.39	0.44
	> 65 years	117	54				
Differentiation	G1	18	9	0.05	0.12	0.14	0.80
	G2	138	69				
	G3	45	22				
	Missing	16					
pTNM	I	135	63	0.37	0.32	0.40	0.37
	II	42	19				
	III	35	19				
	IV	4	2				
	Missing	1					
pT	pT1	68	31	0.76	0.30	0.45	0.73
	pT2	120	56				
	pT3	17	8				
	pT4	11	5				
	Missing	1					
pN	0	157	73	0.32	0.32	0.71	0.37
	1	38	17				
	2	21	10				
	Missing	1					
Tumor size	≤ 3.0 cm	120	56	0.01	0.03	0.76	0.49
	≥ 3.1 cm	96	44				
	Missing	1					
Surgery	Lobectomy	147	68	0.38	0.30	0.41	0.59
	Pulmectomy	26	12				
	Wedge resection	17	8				
	Bilobectomy	17	8				
	Other	10	4				
Tobacco use	Current smoker	74	34	0.22	0.33	0.67	0.86
	Former smoker	129	60				
	Never smoker	14	6				
Packyears	0	14	7	0.13	0.43	0.88	0.42
	1-5	4	2				
	6-20	39	18				
	21-40	105	48				
	41-60	42	19				
	> 60	13	6				
