# Supplementary material for: Expression of S100A4, ephrin-A1 and osteopontin in non-small cell lung cancer
Source: BMC Cancer. 2012 Aug 1;12:333. doi: 10.1186/1471-2407-12-333 (PMC3458900; doi:10.1186/1471-2407-12-333)
Supplement: Additional file 2 — Associations between immunohistochemical expression of cytoplasmic and nuclear S100A4 and selected clinicopathological parameters in adenocarcinomas. [file 1471-2407-12-333-S2.pdf]

Additional file 2

Associations between immunohistochemical expression of cytoplasmic and nuclear S100A4 and selected clinicopathological parameters in adenocarcinomas

			S100A4c				S100A4n				
		Number	Neg/weak	Moderate	Strong	p	Negative	Weak	Moderate	Strong	p
All cases		123	%	%	%		%	%	%	%	
pT stage	pT1	41	25	39	37		7	22	34	37	
	pT2	64	42	31	27		14	41	20	25	
	pT3	9	33	45	22		22	11	45	22	
	pT4	9	22	56	22	0.36	22	45	11	22	0.04
pN stage	pN0	91	27	41	32		9	33	26	32	
	pN1	19	47	37	16		16	37	36	11	
	pN2	13	61	8	31	0.04	38	23	8	31	0.04
pTNM	I	76	26	41	33		8	32	27	33	
	II	21	43	33	24		10	33	38	19	
	III	23	48	30	22		30	35	13	22	
	IV	3	67	0	33	0.04	33	33	0	34	0.01
Tumor size (cm)	< 2.0	36	25	47	28		14	30	28	28	
	2.1-3.0	44	27	32	41		11	25	25	39	
	3.1-5.0	34	44	32	24		17	32	27	24	
	5.1-7.0	6	83	17	0		0	100	0	0	
	>7.0	3	33	67	0	0.02	0	33	67	0	0.23
Packyears	0	12	50	42	8		25	42	33	0	
	1-5	3	67	0	33		33	33	0	34	
	6-20	24	50	33	17		17	41	25	17	
	21-40	58	21	43	36		10	21	33	36	
	41-60	21	43	28	29		10	48	14	28	
	> 60	5	20	20	60	0.02	0	40	0	60	0.01
